# Lipid, Fatty Acid and Energy Density Profiles of White Sharks: Insights into the Feeding Ecology and Ecophysiology of a Complex Top Predator

**DOI:** 10.1371/journal.pone.0097877

**Published:** 2014-05-28

**Authors:** Heidi R. Pethybridge, Christopher C. Parrish, Barry D. Bruce, Jock W. Young, Peter D. Nichols

**Affiliations:** 1 CSIRO Wealth from Ocean Flagship, Division of Marine and Atmospheric Research, Hobart, Australia; 2 Department of Ocean Sciences, Memorial University of Newfoundland, St. John's, Newfoundland, Canada; Scottish Association for Marine Science, United Kingdom

## Abstract

Lipids are major sources of metabolic energy in sharks and are closely linked to environmental conditions and biological cycles, such as those related to diet, reproduction and migration. In this study, we report for the first time, the total lipid content, lipid class composition and fatty acid profiles of muscle and liver tissue of white sharks, *Carcharodon carcharias*, of various lengths (1.5–3.9 m), sampled at two geographically separate areas off southern and eastern Australia. Muscle tissue was low in total lipid content (<0.9% wet mass, wm) and was dominated by phospholipids (>90% of total lipid) and polyunsaturated fatty acids (34±12% of total fatty acids). In contrast, liver was high in total lipid which varied between 51–81% wm and was dominated by triacylglycerols (>93%) and monounsaturated fatty acids (36±12%). With knowledge of total lipid and dry tissue mass, we estimated the energy density of muscle (18.4±0.1 kJ g^−1^ dm) and liver (34.1±3.2 kJ g^−1^ dm), demonstrating that white sharks have very high energetic requirements. High among-individual variation in these biochemical parameters and related trophic markers were observed, but were not related to any one biological or environmental factor. Signature fatty acid profiles suggest that white sharks over the size range examined are generalist predators with fish, elasmobranchs and mammalian blubber all contributing to the diet. The ecological applications and physiological influences of lipids in white sharks are discussed along with recommendations for future research, including the use of non-lethal sampling to examine the nutritional condition, energetics and dietary relationships among and between individuals. Such knowledge is fundamental to better understand the implications of environmental perturbations on this iconic and threatened species.

## Introduction

The white shark, *Carcharodon carcharias*, is considered the most voracious apex predator in temperate marine ecosystems worldwide [1], playing a key role in controlling ecosystem dynamics [2]. Despite this, limited quantitative dietary or energetic data for the species exist. Due to long standing declines in their population, white sharks are protected internationally; listed on Appendix II of the CITES and the Convention of Migratory Species [3]. This makes assessing contemporary biological parameters particularly difficult as investigative methods must be based on a limited number of samples, and be as non-invasive as possible. Consequently, much of our existing dietary knowledge on white sharks has been based on examining deceased sharks taken as incidental bycatch [4] or sharks taken as part of control programs [5]. These data have been supplemented by *in situ* observations of predation at aggregation sites (typically around seal colonies) or scavenging events on whale carcasses [4]–[8] which, although non-invasive, are likely to bias the recording of marine mammals in their diet. This has fueled a general (public) perception that white sharks almost exclusively consume prey comprising these taxa.

Rapid advances in technology, data treatment and other numerical techniques, and our understanding of their tissue biochemistry, are allowing ecologists to gain a more complete understanding of white shark biology. Tracking studies have shown that white sharks engage in broad-scale inshore and offshore migrations [9], [10]. Population genetics have revealed that white sharks exhibit fine-scale spatial structure and low effective population sizes [11]. Stable isotope data of carbon and nitrogen of white shark vertebrae and muscle tissue suggest large ontogenetic and among-individual variation in their foraging strategies [1], [12]. Although not yet applied in studies of white sharks, signature lipid and fatty acid analysis is also gaining momentum to refine our ecological and physiological understanding of sharks [13], [14]. This is mainly because lipids are major sources of metabolic energy and are closely linked to environmental conditions such as prey quality and quantity, and to biological cycles, such as those related to reproduction and migration. More recently, signature fatty acid analysis was validated as a technique to determine dietary changes in a controlled feeding study of Port Jackson sharks [15], and the extraction of fatty acids from muscle tissue biopsies of less than 0.1 g was used to analyze dietary variation in live, threatened manta rays and whale sharks [16]. The successful utilization of signature fatty acids is related to the well known expression ‘you are what you eat’, in that many fatty acids are transferrable from prey to predator with limited modification [17].

The aim of this study was to report the lipid content, lipid class composition and fatty acid profiles of the muscle and liver of white sharks sampled opportunistically from South Australia and New South Wales (central-east Australia). As tissue differences in lipids are well known in other species of shark [18], we compared muscle and liver tissues, and also muscle tissue sampled at different anatomical sites. Lipid content was used to calculate energy density profiles, and is an indicator of an individual's nutritional condition; lipid class composition helped shed light on an individual's physiology; fatty acid profiles were used to infer integrated dietary signatures. A particular focus was placed on examining intra-specific variation and determining the most appropriate way to apply lipid analysis to future ecological studies of white sharks and other protected, large oceanic sharks.

## Materials and Methods

### Ethics statement

All procedures were ratified by independent Animal Ethics Committees, including the New South Wales Department of Primary Industries (NSW DPI) Animal Research Authority (ACEC 12/07) and the Tasmanian Department of Primary Industries, Parks, Water and Environment (ACE 22/2011–2012). In South Australia, field work at the Neptune Islands was carried out under a South Australian S115 ministerial exemption 9902123. In NSW, field work was carried out under a NSW DPI Scientific Collection Permit (P07/0099-3.0 & P07/0099-4).

### Sampling

Twenty one deceased white sharks of various lengths (1.5 to 3.9 m) were sampled opportunistically between 2001 to 2012 in waters off South Australia (SA) and along the New South Wales (NSW) coast, Australia (Table 1). Most specimens were taken either as fisheries bycatch or during the NSW Shark Control Program. The one basking shark included in this study was collected on the beach of Marrawah, north-western Tasmania, in 2012. Sub-samples of muscle (0.05–0.08 g) and liver (0.03–0.05 g) tissues were taken, stored in sterile cryotubes, and kept frozen at −20°C until lipid analysis.

**Table 1:** Collection and biological information of the 21 white sharks (WS) and a single basking shark (BS1) analyzed in this study.

						Tissue analyzed					
ID	Date	Length (m)	Sex	State	Port	M	Mv	Md	Mdf	Mds	L
WS1	17/01/2011	2.25	F	NSW	Maroubra		a				
WS2	23/10/2003	1.85	Unk	NSW	Dee Why			a			
WS3	1/12/2005	1.9	Unk	NSW	Avalon		a				
WS4	24/09/2001	1.74	M	NSW	Stockton			a		b	
WS5	29/01/2003	3.81	M	SA	Port Lincoln		a				
WS7	24/09/2001	1.84	Unk	NSW	Stockton			a			
WS9	30/10/2002	3.9	F	SA	Port Lincoln		a				
WS10	1/01/2005	2.08	M	NSW	Wattamola		a				
WS11	20/02/2013	1.84	F	NSW	Coledale	c	e	b		a	d
WS12	2/05/2012	1.47	F	NSW	Coledale			a			b
WS13	2/05/2013	2.7	M	NSW	Cronulla				a		
WS14	2/05/2012	2.21	M	NSW	Warriewood			b			c
WS15	2/05/2012	2.7	M	NSW	Cronulla		c	a			b
WS16	2/05/2012	2.1	M	NSW	Wollongong			a			b
WS17	2/05/2012	1.78	F	NSW	Coledale			a			b
WS18	16/11/2012	1.9	F	NSW	Bondi			a			
WS19	27/11/2000	3.3	F	SA	Ceduna			a			b
WS21	14/03/2005	2.5	M	NSW	Nth Cronulla		b			a	
WS22	3/10/2002	2.38	M	NSW	Cath Hill Bay		a				
WS23	1/10/2002	2.54	Unk	NSW	Newcastle		a				
WS24	2/12/2002	2.0	M	SA	Port Lincoln		a				
BS1	11/06/2012	3.8	F	TAS	Marrawah			a			b

Sex: F- female, M- male, Unk - sex not identified. State: New South Wales (NSW), South Australia (SA), Tasmania (TAS). Tissues analyzed include the muscle (M), and muscle sampled from the: vertebrae (Mv), dorsal area (Md), dorsal fin not including skin (Mdf), and dorsal fin including the skin (Mds) and the liver (L).

### Lipid content and class composition

Total lipid content was extracted by the modified Bligh and Dyer [19] method using a one-phase CHCl_3_:MeOH:milliQ H_2_O solvent mixture (10∶20∶7.5 mL) which was left overnight. The following day, the solution was broken into two phases by adding 10 mL CHCl_3_ and 10 mL saline milliQ H_2_O (9 g NaCl L^−1^) to give a final solvent ratio of 1∶1∶0.9. The lower layer was drained into a 50 mL round bottom flask and concentrated using a rotary evaporator. The extract was transferred in CHCl_3_ into a pre-weighed glass 2 mL vial. The solvent was blown down under a constant stream of nitrogen gas. The round bottom flask was rinsed twice with CHCl_3_ into the vial. The total lipid extract (TLE) was dried in the vial to constant weight and 1.5 mL of CHCl_3_ added.

Lipid class composition of tissues was determined using an Iatroscan Mark V TH10 thin layer chromatograph coupled with a flame ionization detector (TLC-FID). For each sample, the TLE was spotted and developed in a polar solvent system (70∶10∶0.1 v/v/v hexane:diethyl ether: glacial acetic acid). Samples were also run in a non-polar solvent system (96∶4 v/v hexane:ether) to resolve hydrocarbons (including squalene) from wax esters and diacylglyceryl ethers from triacylglycerols. All samples were run in duplicate along with standard solutions, which contained known quantities of common lipid classes. Chromarods were oven-dried for 10 min at 100°C and analyzed immediately. Peaks were quantified using SIC-480 Scientific Software.

Fatty acid proportions were determined using a direct methylation procedure [20]. Briefly, a small sub-sample (20–50 mg) of wet tissue was transferred to a glass tube, then directly transmethylated in methanol:dichloromethane:concentrated hydrochloric acid (10∶1∶1 v/v) for 2 hours at 80°C. Following the addition of 1.5 mL of Milli-Q water, a known concentration of internal injection standard (23∶0 FAME) was added. After thorough mixing, the tube was centrifuged at 2000 rpm for 5 mins. The upper, organic layer was removed and reduced under a nitrogen gas stream and 0.2 µl from this solution was injected into an Agilent Technologies 7890B gas chromatograph (GC) (Palo Alto, California USA) equipped with an Equity-1 fused silica capillary column (15 m×0.1 mm internal diameter and 0.1 µm film thickness), a flame ionization detector, a splitless injector and an Agilent Technologies 7683B Series auto-sampler. At an oven temperature of 120°C, samples were injected in splitless mode and carried by helium gas. Oven temperature was raised to 270°C at 10°C per min, and then to 310°C at 5°C per min. Peaks were quantified using Agilent Technologies ChemStation software (Palo Alto, California USA). Confirmation of peak identifications was by GC-mass spectrometry (GC-MS), using a column of similar polarity to that described above and a Finnigan Thermoquest DSQ GC-MS system.

### Dry mass and energy density of tissues

Water fraction (WF) of tissue mass was determined by taking weights before and after freeze-drying at −82°C for 48 h. From this, and acquired values of the lipid fraction (LF), we determined the wet/dry mass ratio of each tissue and the proportion that consisted of protein and carbohydrate (PCF_dm_ = 1 - LF_dm_ and PCF_wm_ = 1 - LF_wm_ - WF). Energy density (ED, kJ g^−1^) of wet mass and dry mass of muscle and liver was then calculated using proximate composition data and published calorific values of 39.3 kJ g^−1^ for lipid and 17.8 kJ g^−1^ for protein and carbohydrates [21] with the following equations:
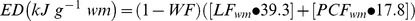


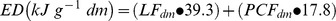



### Multivariate statistics

We used PRIMER (Plymouth Routines in Multivariate Ecological Research), a software package for analyzing multivariate ecological data, to assess the groupings and investigate relationships within the fatty acid data set. Specifically, we used parametric, principal component analysis (PCA) and non-parametric cluster analysis.

### Prey-predator and interspecies fatty acid comparisons

To assist in assessing broad scale food web groupings, a number of established fatty acid markers, or trophic indices, were calculated. These included 16:1ω7/16:0 and 22:6ω3/20:5ω3 to separate diatom versus dinoflagellate based energy channels [22]; 18:1ω9/18:1ω7 which have been linked to trophic position [23]; and 20:4ω6/20:5ω3 and ω6/ω3 which are nutritional condition indices, and may also provide information on benthic-derived inputs.

Of the total of fifty fatty acids detected in white sharks tissues, twenty two (with averages >0.05% of total fatty acids) were used in multivariate analyses to compare white shark muscle and liver profiles to other oceanic and coastal shark taxa with known dietary differences. Specifically, the mean fatty acid profiles of each of white shark muscle and liver, grouped according to cluster analyses, were compared to profiles of *in situ* caught school, gummy and broadnose sharks (*Galeorhinus galeus*, *Mustelus antarcticus*, *Notorynchus cepedianus*; [24], Australian catshark and shortnose spurdog (*Figaro boardmani* and *Squalus megalops*, [18], Port Jackson shark (*Heterodontus portusjacksoni* [15]), and liver profiles of tiger shark (*Galeocerdo cuvieri*) sampled off New South Wales in 2000 [25] and white shark sampled off South Africa [26].

## Results and Discussion

### Water, lipid content and class composition

White shark muscle was high in water (75.4±2.4%) and had a wet to dry mass ratio of 4.1±0.4 (Table 2). Muscle was low in total lipid content (consistently <0.9% wm) and varied marginally between different body sections (average coefficient of variation was only 6%). The proportion of wet mass that was protein and carbohydrate was 23.9%. The energetic density of white shark muscle was estimated at 4.5±0.5 kJ g^−1^ wm (Table 2). Phospholipids dominated the total lipid fraction (92.4±4.8%) with only small relative levels of sterols (6.1±4.1%) and free fatty acids (1.4±0.7%) present. In the only other study that has reported lipid components in white shark muscle [27], similar lipid content was observed in specimens (of unknown sizes) collected off South Africa (6.1±3.3 mg g^−1^ wm). In other shark species, similar total lipid content has been detected; typically accounting for less than 1% wm [28], [18]. In another study more variable total lipid content was reported in muscle for 11 species of shark in the Caribbean (0.4–11% dm) with higher total lipid content occurring in hammerhead, seven-gills and six-gills [29]. The present study is the first to report the muscle lipid class composition of white sharks or any other large oceanic shark, but the observed profiles are comparable to those of demersal sharks [18] which have high relative levels of structural components (phospholipids) and nil to low levels of energy storage (neutral) lipids.

**Table 2:** Biochemical parameters of white shark tissues collected from temperate waters off south east Australia.

Parameter			muscle	liver
Water content	%	wm	75.4±2.4	20.0±5.4
Wet/dry ratio	-		4.1±0.4	1.2±0.04
Total lipid content	%	dm	2.9±0.6	75.9±11.5
	%	wm	0.7±0.1	61.0±13.3
Total fatty acids	%	wm	0.5±0.1	13.0±0.1
Protein & carbohydrates∧	%	dm	97.2±1.0	24.1±6.2
Energy density	kJ g^−1^	dm*	18.4±0.1	34.1±3.2
	kJ g^−1^	wm*	4.5±0.5	27.3±2.8
Lipid class composition				
Triacylglycerols (TAG)	%	wm	0.0±0.1	96.3±0.9
Phospholipids (PL)	%	wm	92.4±4.8	1.9±1.0
Sterols (ST)	%	wm	6.1±4.1	0.4±0.4
Wax esters (WE)	%	wm	0.0±0.0	1.5±0.1
Free fatty acids (FFA)	%	wm	1.4±0.7	0.1±0.2

∧Estimated by subtracting the lipid fraction from 100%.

*For comparative studies, 1 kilocalorie = 4.184 kilojoules (kJ).

Values are in dry and wet mass (dm and wm).

Liver had a wet to dry ratio of 1.2±0.1 (Table 2) and was lower in water content (16–24%) than that reported for the Atlantic sharpnose shark (25–70% [30]), the only other shark species for which such data is published (to the authors' knowledge). Total lipid content varied between 51.6–70.4% wm (67.8–84.1% dm) and was dominated by triacylglycerols (96.3±0.9%), followed by phospholipids (1.9±1.0%), wax and steryl esters (1.5±0.1%), sterols (0.4±0.4%), and free fatty acids (0.1±0.2%). The proportion of wet liver that was protein and carbohydrates was 4.1% and the liver-specific energy density was estimated at 27.3±2.8 kJ g^−1^ wm (Table 2). Total lipid content and lipid class composition of the liver of other large oceanic sharks has seldom been measured, and has yet to be reported for white sharks. In other oceanic sharks, total lipid content has been reported to be highly variable both inter-specifically (10 to 90% wm; [29], [31], [25]) and intra-specifically [32]. Nichols et al. [25] found that more than half the 17 species of pelagic sharks collected in northern Australian waters had liver lipid contents greater than 40% including the tiger shark, milk shark, school, gummy, whaler and blue sharks. Another study [27] reported very few inter-specific differences in total lipid content between blacktip and spinner sharks, but large differences between samples collected in the Atlantic and Indian Oceans. Furthermore, Davidson & Cliff [32] found large seasonal differences in grey nurse shark liver mass (10–16 kg) and lipid concentrations (326–572 mg g^−1^ wm) and related this to their migration and reproductive cycles. For most oceanic shark species analyzed, triacylglycerol seems to be the dominant class of lipid accounting for >90% total lipid [25], [33]. In contrast, liver lipids of sharks living at more than 200 m depth are dominated by diacylglyceryl ethers and the isoprenoid hydrocarbon squalene [18].

Storage of large amounts of lipids which are high in energy and low in density are likely to assist white shark movement patterns [34]. Triacylglycerols (at 1 atmospheric pressure and 20°C) have an energy density of 0.93 g L^−1^ and the buoyancy in sea water (0.0103 g^−1^) is +0.095 mL^−1^ lipid [35]. Because triacylglycerols are used for metabolism at higher rates than wax esters, diacylglyceryl ethers and squalene [36], they play a less important role in buoyancy and instead their major function is energy storage which can be used for energetically exhausting activities such as migration, reproduction or during extended periods of low prey availability. As both squalene and diacylglyceryl ethers, which have lower energy densities of 0.86 and 0.89 g L^−1^, respectively [35], are more abundant in deep-sea sharks [18], the amount of lift generated in the white shark per unit weight of liver oil is lower than in deep-sea sharks, but high enough to achieve neutral buoyancy in seawater. Our results show that liver lipid reserves are unlikely to remain constant over time as was assumed by Raye et al. [34] and therefore, buoyant weight of the shark does not necessarily indicate a change in lipid volume. However, changes in lipid class composition could account for differences in buoyant mass.

### White shark energetics

The estimated energy density of white shark muscle was noticeably lower than that of the liver (mean values: 4.5 kJ g^−1^ wm and 27.3 kJ g^−1^ wm, respectively, Table 2). This represents the total available energy from these tissues and supports the function of liver as an energy reserve. From the available liver weights of sharks analyzed in this study, liver was found to account for between 8.2–16.2% of total body mass. Using an estimate for muscle of 45% of total body mass, and the mean liver value of 12.2%, we calculated the muscle and liver energy content for a 428 kg individual to be 867 MJ wm and 1425 MJ wm, respectively. Few studies have investigated the energy density or content of elasmobranches. Based on bomb calorimeter, seasonal and inter-annual differences in the liver-specific energy content (which varied between 26.8 to 34.8 kJ g^−1^ dm) and energy content (590 to 4550 kJ dm) were observed in the Atlantic sharpnose shark in the Gulf of Mexico [36]. The only other reports (to the authors' knowledge) of energy density in shark tissue used a value of 5.41 kJ g^−1^ wm [37], [38], based on consumption estimates given for lemon shark by Cortés & Gruber [39]. This value seems to be grossly underestimated for larger oceanic sharks as shown by the present study.

In comparison to other fish (demersal and pelagic) and mammals, white shark liver has a higher energy density, and therefore capacity to store energy. Drazen [40] reported high variability in the muscle and liver energy density between 22 species of demersal fish in the Pacific Ocean. They found that energy densities were consistently lower (between 1.6 and 13.6 times, and on average 4.3 times) in the muscle (1.2–5.5 kJ g^−1^ wm) than liver (4.2–17.9 kJ g^−1^ wm) with benthopelagic species, primarily gadiforms, having significantly larger lipid-rich livers than benthic species. In a comparative study of the energy density of 39 pelagic forage fishes [41], a five-fold difference (2.0–10.8 kJ g^−1^ wm fish) was reported with small, fast maturing species having higher relative values than those species maturing at a larger size. Energy density of muscle and liver of Antarctic fishes were respectively lower (21–25 kJ g^−1^ dm) and higher (25–30 kJ g^−1^ dm, [42]) than in the white sharks analyzed in the present study. Whereas sharks store energy in their livers, mammals store energy in adipose tissue with whale blubber having an estimated energy density of 27.5 to 30.6 kJ g^−1^ [43]. Such comparisons suggest that white sharks have much higher energy storage needs than whales or demersal and pelagic fishes. This is likely related to their high energetic requirements related to undertaking long migrations, reproduction (including courtship) and foraging.

A recent study [44] estimated daily energetic expenditure of adult white sharks at a seal colony off South Australia to be 28,200 kJ d^−1^. Our study adds information on the static energy available in the muscle and liver of white sharks and gives additional information on their energy budget and allocation strategies. Such information is vital for the calibration and validation of bioenergetics models at individual, population and ecosystem scales. These models, which describe the mass balance relationship between energy acquired, transformed and allocated to organism productivity (growth, reproduction and survival), are increasingly being used to assist fisheries and conservation management [45]. As lipid content is the primary determinant of energy density, estimating tissue-specific energy density and content based on partial proximate composition is faster and cheaper than traditional bomb calorimetry. As lipid content variations are food-dependent, they can be directly related to body and energetic condition and are likely to be a robust way to predict features of their population dynamics, such as reproductive potential and ability to sustain long-distance migrations. Indeed, positive associations between recruitment and total lipid energy have been proposed as a robust proxy for total egg production by fish stocks where 2.12 kJ of liver energy was found to be proportional to 1000 eggs [46]. Future research should seek to examine if a similar relationship and proxy approach can be reliably used for elasmobranches.

### Fatty acid profiles

A total of fifty fatty acids were identified in the white shark tissues analyzed (Table S1 and S2), with thirty one fatty acids identified in relative levels greater than 0.1% (Table 3). White shark muscle was proportionally dominated by polyunsaturated fatty acids (PUFA, 35±11%) and saturated fatty acids (SFA, 35±6%), with slightly lower levels of monounsaturated fatty acids (MUFA, 27±10%). Dominant fatty acids (>5%) in decreasing order of relative importance included: 16:0, 22:6ω3, 18:0, 18:1ω9, 20:4ω6 and 18:1ω7. Limited variation in fatty acid proportions was found among muscle sampled at different anatomical sites (Table 1) with the mean coefficient of variation typically less than 16%. Greatest differences in fatty acid profiles were observed between muscle tissue taken from the vertebrae and from the dorsal fin and skin. White shark liver was dominated by MUFA (36±12%) followed by SFA (31±2%) and PUFA (30±12%). Dominant fatty acids in decreasing order of relative importance were similar, with the exception of the lower (twofold) levels of 20:4ω6, to those reported in the muscle and included: 16:0, 18:1ω9, 22:6ω3, 18:0, 16:1ω7 and 18:1ω7.

**Table 3:** Fatty acid distribution of white shark muscle and liver (mean area % of total fatty acids ± standard deviation, and the coefficient of variation %) sampled off south and eastern Australia.

	Muscle			Liver		
n	21			7		
Length	1.74–3.9			1.84–3.3		
14:0	0.81	±0.66	82%	2.59	±1.90	73%
15:0	0.22	±0.15	69%	0.46	±0.30	66%
16:0	18.55	±3.48	19%	18.42	±2.29	12%
17:0	0.64	±0.22	34%	0.70	±0.20	29%
18:0	13.79	±3.64	26%	8.70	±3.93	45%
16:1ω9	0.44	±0.45	102%	0.51	±0.18	36%
16:1ω7	2.13	±1.81	85%	6.72	±5.72	85%
17:1ω8+a17:0	0.63	±0.37	58%	0.78	±0.32	41%
17:1	0.34	±0.31	93%	0.10	±0.09	91%
18:1ω9	11.90	±4.38	37%	16.19	±5.49	34%
18:1ω7	6.25	±1.50	24%	5.96	±1.16	19%
19:1	0.39	±0.11	28%	0.27	±0.06	21%
20:1ω11	0.14	±0.17	127%	0.37	±0.29	79%
20:1ω9	1.94	±0.91	47%	2.27	±1.33	59%
20:1ω7	0.23	±0.12	54%	0.33	±0.16	47%
22:1ω11	0.11	±0.16	138%	0.29	±0.21	74%
22:1ω9	0.45	±0.32	70%	0.62	±0.30	48%
24:1ω9	1.39	±2.26	163%	0.56	±0.47	83%
18:2ω6	0.70	±0.23	34%	0.98	±0.29	30%
20:4ω6 (AA)	9.22	±3.93	43%	4.86	±5.42	111%
20:5ω3 (EPA)	1.63	±1.05	64%	2.31	±0.81	35%
20:4ω3	0.17	±0.12	66%	0.32	±0.14	45%
22:5ω6	0.73	±0.42	57%	0.77	±0.22	29%
22:6ω3 (DHA)	15.52	±6.68	43%	13.62	±6.34	47%
22:4ω6	2.44	±1.17	48%	1.65	±1.24	75%
22:5ω3	2.75	±1.40	51%	3.40	±1.02	30%
**Σ SFA**	**34.70**	**±5.71**	**16%**	**31.36**	**±2.34**	**7%**
**Σ MUFA**	**27.08**	**±9.60**	**35%**	**35.85**	**±11.91**	**33%**
**Σ PUFA**	**34.48**	**±12.19**	**35%**	**29.70**	**±11.83**	**40%**
Σ ω3 PUFA	20.36	±8.60	42%	20.43	±6.99	34%
Σ ω6 PUFA	13.59	±4.90	36%	8.75	±6.46	74%
i15:0	0.33	±0.18	54%	0.42	±0.36	85%
i16:0	0.21	±0.15	71%	0.32	±0.29	92%
i17:0	0.95	±0.39	41%	0.62	±0.09	14%
16:0 FALD (75)	1.37	±0.78	57%	0.93	±0.77	83%
18:0 FALD (75)	0.43	±0.59	136%	0.21	±0.13	61%
Σ iso-SFA	1.70	±0.76	44%	1.72	±0.91	53%
Σ branched FA	2.05	±1.21	59%	1.36	±0.92	67%
**Σ other (<0.2%)**	**2.94**	**±1.40**	**48%**	**3.77**	**±1.08**	**29%**
**Trophic markers**						
16:1ω7/16:0	0.11	±0.10	85%	0.35	±0.31	87%
20:5ω3/22:6ω3	0.12	±0.10	86%	0.21	±0.13	62%
16:0/18:0	1.45	±0.51	35%	2.49	±1.01	40%
18:1ω9/18:1ω7	1.90	±0.59	31%	2.71	±0.85	31%
14:0+16:1ω7+20:5ω3	4.57	±2.84	62%	11.62	±7.62	66%
PUFA/SAT	1.04	±0.43	41%	0.96	±0.40	42%
20:4ω6/20:5ω3	11.95	±14.97	125%	2.59	±3.47	134%

SFA – saturated fatty acids, MUFA – monounsaturated fatty acids, PUFA – polyunsaturated fatty acids. The suffix *i* denotes branched fatty acids from the *iso*-series. FALD - fatty aldehyde analysed as dimethyl acetal.

Other fatty acids (that accounted for <0.2% of total fatty acids) are included in Table S1.

Data presented are for 31 components, with a cut off of 0.2%. For full fatty acid profiles of individual samples, see Table S1.

Muscle fatty acid profiles of white shark in South Africa [25]were similar to those reported in this study with comparable SFA (34.8±7.0%), MUFA (25.3±6.5%), and PUFA (34.5±12.2%PUFA) dominated by 22:6ω3, 20:4ω6, 22:4ω6. Similar to our results, no significant differences in fatty acid profiles of muscle taken from different anatomical sites were found in other large oceanic sharks, including tiger and white sharks sampled off South Africa [25]. Davidson & Cliff [47] reported similar liver fatty acid profiles for white sharks collected off South Africa with PUFA accounting for 26.6%. Long-chain (≥C_20_) polyunsaturated fatty acids have also been reported in large concentrations in the liver of other large, oceanic shark species [26], [23], [24]. In the present study, very high levels of 20:4ω6 were found in white shark muscle (9.2%) with moderate levels in the liver (4.9%) which are within the very upper range of that reported in other oceanic and predatory shark species [47], [25], [22], [16]. Such variability could be related to differences in diet, with higher relative levels representative of benthic/coastal inputs. Another study [48] found that 20:4ω6 levels increased with size in juvenile bull sharks and low relative levels were likely to infer essential fatty acid deficiency.

**Figure 1:**
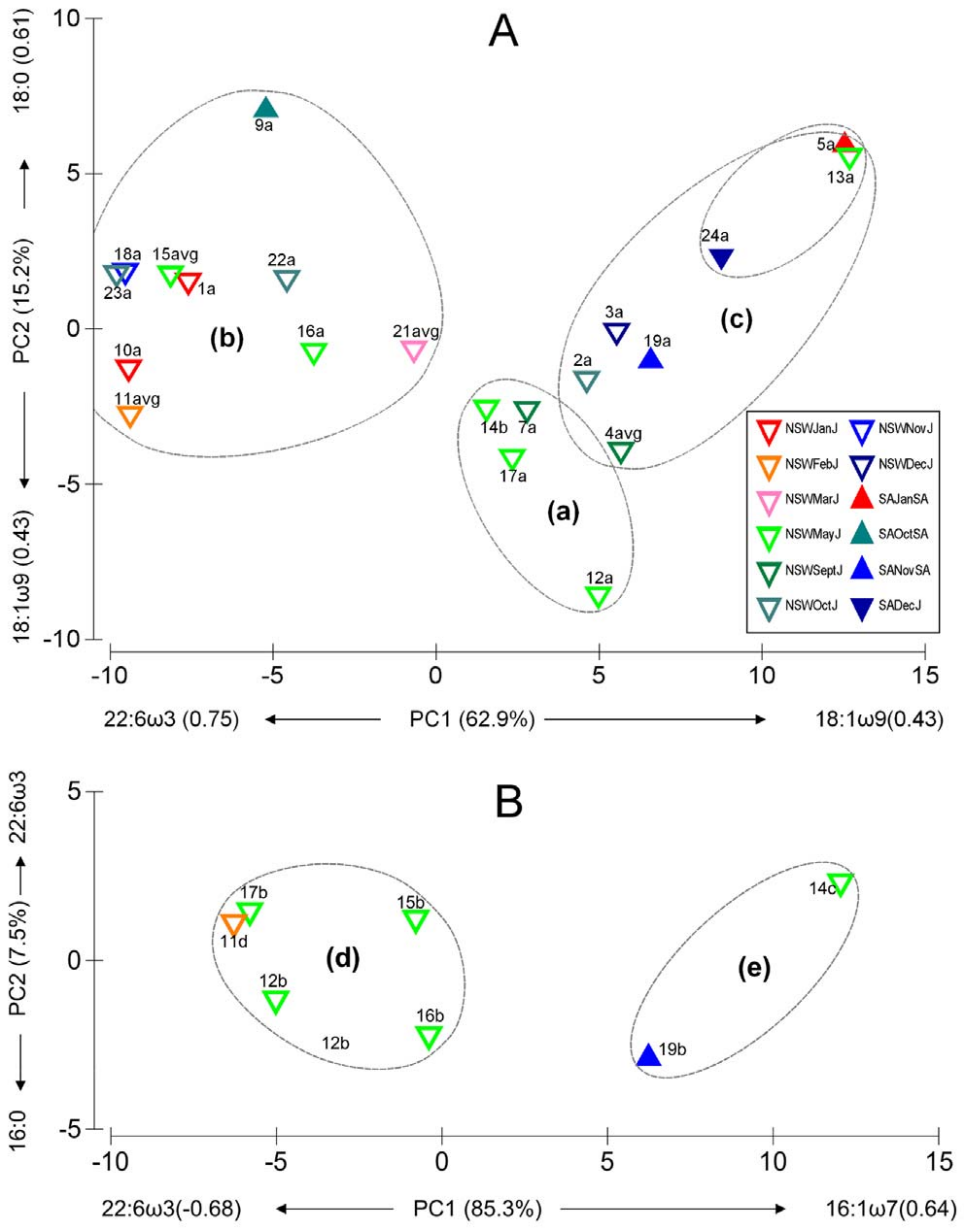
Principal component analysis (PCA) of the fatty acid profiles of juvenile (J) and sub-adult (SA) white shark (A) muscle, and (B) liver collected from New South Wales (NSW) and South Australia (SA) during various months and years. Eigenvalues in brackets represent the percent variance explained by each axis (PC1 and PC2). Fatty acids labeled on each of the axes represent the main coefficients (or eigenvectors) contributing to each PC. Black lines represent groups that have more than 80% similarity based on non-parametric cluster analysis complete linkages. Sample codes are listed in Table 1.

In addition to the large differences occurring between the fatty acid profile of the muscle and liver, we detected large variations among individual sharks (Fig. 1 and Table S1). In the muscle, three clear groups of sharks were apparent based on 80% similarity of a non-parametric cluster analysis: group A consisted of four sub-adult sharks collected in New South Wales in May 2012 and September 2001 which had moderate proportions of 22:6ω3 and low 18:0; group B separated nine sharks of various lengths collected from various sampling months, years (2002, 2005, 2011–2013) and sites due their higher levels of 22:6ω3 and 18:0; while group C consisted of seven sharks high in 18:1ω9 and 18:0 (Fig. 1A). No single biological (size or maturation status) or environmental factor (collection site and month) stood out in the separation of the groups with juvenile and sub-adult sharks in all three groups, and larger (>3 m) sharks from South Australia identified in both groups B and C, although with noticeably higher levels of 18:0 (Fig. 1A). For the seven white shark liver samples, two groups separated based on 80% similarity with one group (D) having higher levels of 22:6ω3, 20:1ω9, and 22:5ω3, while the other group (E) had higher levels of 16:1ω7 and 14:0 (Fig. 1B). If we separated the two PCA axes at the central point, then only the liver sample of one large (adult >3 m female) shark collected from South Australian waters separates from the smaller (sub-adult) sharks collected from east Australia due to high levels of 16:0 and 16:1ω7.

### Dietary inferences

Large intra-specific differences in fatty acid profiles of tissues have been experimentally shown to reflect an organism's diet at different temporal scales [13] and these differences can be directly related to its habitat utilization, nutritional condition and reproductive cycles [11], [12], [16], [49]. Although caution should be taken when interpreting tissue-specific fatty acid profiles due to physiological factors that are not yet well understood in elasmobranchs [16], in this study the fatty acids responsible for separating muscle and liver groups on the PC1 axes were consistently 22:6ω3, 18:1ω9, and 16:1ω7 (Fig. 1). These differences are likely to reflect varying contributions of mammalian blubber and fish/cephalopods to the diets of individual white sharks, as blubber is typically high in 18:1ω9, 16:1ω7, and 20:5ω3 [50], whilst fish and cephalopods are higher in 22:6ω3, 16:0, 18:0 [22], [16]. Previous dietary studies of white sharks show clear ontogenetic dietary differences between juveniles (<3 m, which commonly feed on large fish such as mulloway, salmon, snapper, redfish), and sub-adult (>3.0–3.6 m for males and >3.0–4.8 m for females) and adult (>3.6 m for males and >4.8 m for females) sharks which also feed on fur seals and occasionally on whales [8].

No clear separation among juvenile and adult sharks was found in the present study. This is likely related to the fact that individuals were caught over a long and variable sampling period (throughout the year between 2000 and 2006 and 2011–2013, with a maximum of 6 caught in any one year) and the animals were from a large size range (1.5–3.9 m). This variation, in association with the fact that white sharks engage in broad-scale inshore and offshore migrations [7], [8], means that intra-specific variation should be high. Future studies should seek to detail the fatty acid profiles of dominant prey species of white sharks and target research efforts on individuals with known diets of mammals such as those that occur for extended periods around seal colonies. Furthermore, in association with an increase in the use of telemetry and satellite tracking on white sharks [51], future work should combine telemetry with signature lipid analyses, through the recently developed and applied ability for researchers to obtain tissue biopsies of as little as 0.1 g [14] while placing tracking devices. For energetics studies, we recommend taking a very small (<0.01 g) biopsy of liver tissues over muscle, as unlike teleosts and mammals, sharks do not have adipose tissue and the liver is the main site for lipid storage and synthesis [52]. Our results show that muscle or dermal tissue can be taken from various anatomical sites as limited differences in their fatty acid profiles were observed. Future work should also seek to analyze fatty acid profiles of plasma, which is being increasingly withdrawn and used for genetic work. As shown by dietary studies of mammals [53], [54], knowledge of the fatty acid profiles of white shark plasma will assist the acquisition of dietary data (on a shorter time scale to the muscle and liver) and our current understanding of the cellular uptake and transport of lipids.

**Figure 2:**
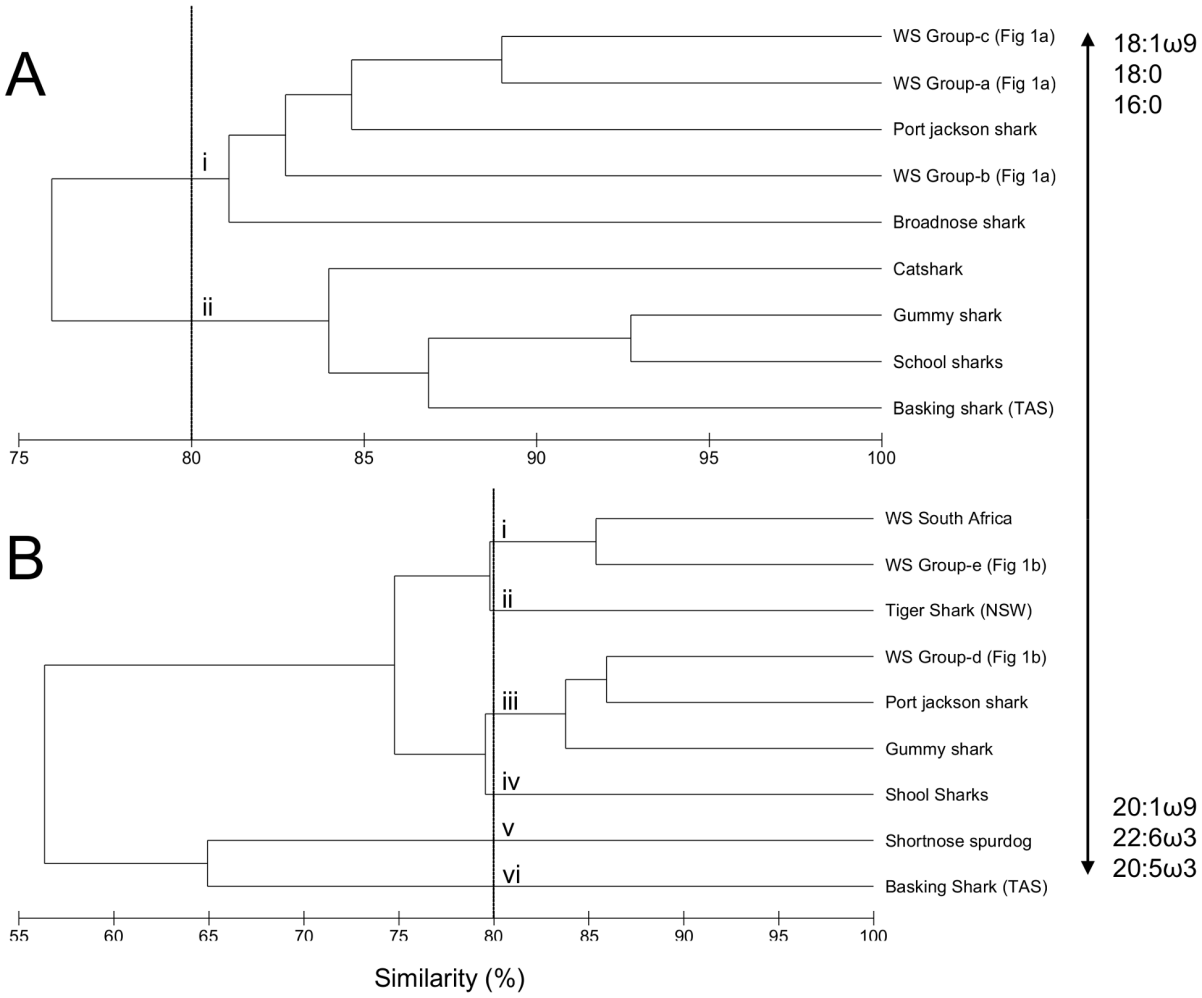
Dendrogram of cluster analysis (group averages) based on a Bray-Curtis similarity matrix for comparison of the fatty acid composition of the (A) muscle and (B) liver of white shark (WS) groups (identified in this study, Fig. 1) and from published data of white sharks collected off South Africa and to other shark species collected in Australian waters.

Inter-specific comparisons can assist in the interpretation of fatty acid data and making dietary inferences, particularly where sample size is low [11], [16], such as in the present study. We therefore used non-parametric cluster analyses of the fatty acid profiles of muscle and liver of white shark groups identified in Fig. 1 and groupings with other shark species in Fig. 2. For the muscle tissue, two clear groups (containing individuals with >80% similarity) were identified with all white sharks identified in Fig. 1 grouping together with Port Jackson and broadnose sharks.

Cluster analyses of the liver fatty acid profiles showed a higher magnitude of dissimilarity between sharks than that in the muscle, with an 80% resemblance cut-off identifying six separate groups (Fig. 2B). White sharks identified in group E (Fig. 1) showed high similarity to the profiles of white sharks collected off South Africa and tiger shark from north Australia, with the latter being known top predators of mid-trophic fish, elasmobranchs, cephalopods, mammals and seabirds [55]. Group D white sharks grouped with Port Jackson sharks. For muscle and liver, the fatty acids of basking shark, known planktivores [56], were the most dissimilar to white sharks. The liver fatty acid profiles of gummy, school and spurdog sharks, all prominent pelagic fish feeders [57], [11] were also dissimilar to the acid profiles of white sharks.

The close similarity between muscle profiles of white shark in the present study and broadnose shark is fitting; the latter is a dominant predator in coastal systems, commonly consuming mammalian blubber and small elasmobranchs including spurdogs, school and gummy sharks [58]. The grouping with the muscle and liver of Port Jackson shark, a tertiary consumer of largely molluscs, teleosts and cephalopods [59], was surprising and may suggest a high intake of these benthic and coastal prey, or alternatively (and most likely) a high consumption of these or similar shark species. The fatty acids mostly responsible for grouping Port Jackson and white sharks were those that are known to reflect trophic position, 16:0 and 18:1ω9 [21], and that are abundant in dinoflagellates (22:6ω3 [20]). In comparison with large predatory fish such as albacore and skipjack tuna [60], white sharks have higher levels of 20:4ω6, 22:4ω6 and 18:1ω7 fatty acids. Collectively, these results suggest that white sharks are more of a generalist species, including benthic/coastal food-chain linkages, than a mammal specialist.

Of particular interest in understanding the diets of white sharks is the contribution of oceanic relative to coastal prey. In a study of pelagic fishes in oceanic waters off eastern Australia, mako shark had the highest nitrogen isotopic value indicating their position as top predator in the system [61]. White sharks are known to move between coastal and oceanic waters, in some cases travelling large distances within and between oceanic basins [7], [8]. Whether these migrations are driven by changes in food, reproductive cycles or other reasons is yet to be determined, although the high energetics related to them means that maintaining lipid reserves in the liver is a crucial, and perhaps, regulatory component. We are hopeful that signature lipid and fatty acid analyses can assist in obtaining a better understanding of the trophic ecology of white sharks.

## Conclusions

In this study we used lipid analyses to directly extract energetic and feeding related information on white sharks. We found that while the muscle is a site of structural energy, the liver has an even higher energy density than whale blubber and serves as a storage unit to fuel white shark migrations, growth and reproduction. Among-individual variation in fatty acid profiles suggest individuality in foraging strategies with some sort of a gradient between dominant or partial consumption of fish and mammals. Such information, although currently very limited in elasmobrachs, is required for the development of bioenergetics-based models including dynamic energy budget models and ecosystem models increasingly used in fisheries and conservation management. Indeed, measuring total lipid energy is appealing because lipids represent a common currency, linking food quality and quantity to many aspects of fish nutrition, reproduction and population dynamics. Future studies should include targeted sampling and utilization of this novel approach on live specimens, along with ongoing telemetry studies, to explore the relative contributions of biological and environmental factors influencing intra-specific variation. Such data will allow us to better understand the ecology of white shark and predict how they might respond to environmental perturbations.

## Supporting Information

Table S1 Muscle full fatty acid profiles of individual white sharks and a single basking shark (BS1) analyzed in this study.
(DOCX)

Table S2 Liver full fatty acid profiles of individual white sharks and a single basking shark (BS1) analyzed in this study.
(DOCX)
