# Supplementary material for: Lipid, Fatty Acid and Energy Density Profiles of White Sharks: Insights into the Feeding Ecology and Ecophysiology of a Complex Top Predator
Source: PLoS One. 2014 May 28;9(5):e97877. doi: 10.1371/journal.pone.0097877 (PMC4037211; doi:10.1371/journal.pone.0097877)
Supplement: Table S1 — Muscle full fatty acid profiles of individual white sharks and a single basking shark (BS1) analyzed in this study. (DOCX) [file pone.0097877.s001.docx]

**Table S1.** Muscle full fatty acid profiles of individual white sharks and a single basking shark (BS1) analyzed in this study.

| Samples | 1a | 2a | 3a | 4 all | 5a | 7a | 9a | 10a | 11all | 12a | 13a | 14b | 15all | 16a | 17a | 18a | 19a | 22a | 21all | 23a | 24a | BS1a |
| --- | --- | --- | --- | --- | --- | --- | --- | --- | --- | --- | --- | --- | --- | --- | --- | --- | --- | --- | --- | --- | --- | --- |
| 14:0 | 0.31 | 1.07 | 0.67 | 0.54 | 0.69 | 0.67 | 0.48 | 0.33 | 0.43 | 1.08 | 1.22 | 1.39 | 0.39 | 0.37 | 0.91 | 0.47 | 0.96 | 0.53 | 0.35 | 0.21 | 0.84 | 0.79 |
| 15:0 | 0.08 | 0.33 | 0.24 | 0.26 | 0.16 | 0.22 | 0.00 | 0.14 | 0.13 | 0.26 | 0.43 | 0.21 | 0.08 | 0.16 | 0.26 | 0.12 | 0.20 | 0.12 | 0.14 | 0.02 | 0.27 | 0.15 |
| 16:0 | 16.22 | 23.01 | 25.27 | 23.06 | 12.45 | 18.89 | 16.54 | 17.88 | 15.59 | 18.88 | 21.87 | 15.96 | 16.90 | 19.85 | 20.37 | 15.13 | 20.83 | 16.91 | 22.83 | 17.52 | 18.03 | 17.83 |
| 17:0 | 0.48 | 0.91 | 0.91 | 0.88 | 0.32 | 0.83 | 0.38 | 0.72 | 0.56 | 0.64 | 0.96 | 0.51 | 0.48 | 0.93 | 0.80 | 0.45 | 0.42 | 0.42 | 0.61 | 0.36 | 0.57 | 0.40 |
| 18:0 | 15.45 | 13.77 | 16.98 | 14.06 | 13.06 | 12.83 | 22.26 | 13.84 | 11.85 | 8.87 | 19.39 | 11.87 | 15.65 | 15.16 | 12.02 | 15.49 | 14.57 | 16.18 | 15.93 | 15.57 | 14.85 | 12.99 |
| 19:0 | 0.15 | 0.23 | 0.23 | 0.29 | 0.23 | 0.24 | 0.14 | 0.15 | 0.13 | 0.17 | 0.31 | 0.13 | 0.13 | 0.21 | 0.16 | 0.13 | 0.19 | 0.13 | 0.17 | 0.11 | 0.17 | 0.05 |
| 20:0 | 0.14 | 0.14 | 0.16 | 0.07 | 0.33 | 0.10 | 0.16 | 0.08 | 0.11 | 0.10 | 0.38 | 0.16 | 0.14 | 0.15 | 0.12 | 0.11 | 0.13 | 0.11 | 0.09 | 0.09 | 0.26 | 0.06 |
| 22:0 | 0.22 | 0.31 | 0.14 | 0.08 | 0.77 | 0.16 | 0.21 | 0.10 | 0.16 | 0.13 | 1.35 | 0.19 | 0.20 | 0.21 | 0.11 | 0.17 | 0.13 | 0.14 | 0.14 | 0.16 | 0.49 | 0.05 |
| 24:0 | 0.12 | 0.12 | 0.09 | 0.01 | 0.56 | 0.07 | 0.11 | 0.05 | 0.09 | 0.04 | 0.52 | 0.08 | 0.09 | 0.09 | 0.07 | 0.07 | 0.07 | 0.09 | 0.06 | 0.03 | 0.35 | 0.00 |
| 16:1ω9 | 0.21 | 0.52 | 0.30 | 0.32 | 2.22 | 0.38 | 0.13 | 0.19 | 0.28 | 0.60 | 0.66 | 0.30 | 0.24 | 0.22 | 0.39 | 0.16 | 0.24 | 0.30 | 0.25 | 0.12 | 1.16 | 0.49 |
| 16:1ω7 | 1.01 | 1.89 | 1.52 | 1.54 | 1.94 | 2.14 | 0.67 | 0.63 | 0.98 | 2.83 | 3.21 | 5.55 | 1.09 | 1.25 | 2.07 | 0.86 | 4.66 | 1.75 | 1.45 | 0.74 | 2.71 | 1.51 |
| 17:1ω8+a17:0 | 0.31 | 0.83 | 0.64 | 0.82 | 0.82 | 0.97 | 0.22 | 0.45 | 0.45 | 0.86 | 1.85 | 0.51 | 0.33 | 0.57 | 0.69 | 0.29 | 0.54 | 0.28 | 0.56 | 0.23 | 0.69 | 0.20 |
| 17:1 | 0.52 | 0.25 | 0.18 | 0.16 | 0.99 | 0.33 | 0.42 | 0.21 | 0.21 | 0.22 | 0.92 | 0.31 | 0.20 | 0.19 | 0.16 | 0.29 | 0.13 | 0.37 | 0.23 | 0.29 | 1.24 | 0.19 |
| 18:1ω9 | 8.13 | 13.12 | 12.01 | 15.94 | 15.87 | 14.36 | 6.74 | 7.32 | 8.51 | 18.68 | 13.58 | 13.78 | 7.41 | 9.21 | 14.14 | 6.91 | 12.93 | 8.72 | 9.52 | 6.17 | 14.84 | 10.13 |
| 18:1ω7 | 4.95 | 6.85 | 7.24 | 7.70 | 6.24 | 7.21 | 4.63 | 5.20 | 5.39 | 7.78 | 6.08 | 6.83 | 4.99 | 6.56 | 6.93 | 4.94 | 10.88 | 5.89 | 7.50 | 4.68 | 6.13 | 5.34 |
| 18:1ω5 | 0.09 | 0.14 | 0.14 | 0.28 | 0.11 | 0.19 | 0.08 | 0.01 | 0.13 | 0.26 | 0.15 | 0.19 | 0.06 | 0.17 | 0.23 | 0.08 | 0.24 | 0.09 | 0.15 | 0.06 | 0.11 | 0.27 |
| 19:1 | 0.35 | 0.43 | 0.56 | 0.46 | 0.60 | 0.48 | 0.29 | 0.46 | 0.32 | 0.36 | 0.51 | 0.34 | 0.33 | 0.51 | 0.36 | 0.27 | 0.40 | 0.34 | 0.50 | 0.24 | 0.55 | 0.20 |
| 20:1ω11 | 0.00 | 0.18 | 0.13 | 0.30 | 0.00 | 0.23 | 0.00 | 0.00 | 0.18 | 0.06 | 0.00 | 0.00 | 0.02 | 0.11 | 0.31 | 0.00 | 0.00 | 0.00 | 0.00 | 0.00 | 0.00 | 0.27 |
| 20:1ω9 | 0.84 | 2.28 | 2.08 | 2.85 | 2.47 | 2.88 | 1.22 | 1.64 | 1.44 | 4.16 | 1.74 | 1.70 | 0.97 | 1.54 | 2.35 | 0.74 | 1.67 | 1.06 | 1.36 | 0.66 | 2.04 | 3.70 |
| 20:1ω7 | 0.12 | 0.18 | 0.15 | 0.21 | 0.55 | 0.21 | 0.17 | 0.13 | 0.16 | 0.29 | 0.34 | 0.32 | 0.11 | 0.26 | 0.22 | 0.09 | 0.26 | 0.18 | 0.18 | 0.08 | 0.42 | 0.18 |
| 22:1ω11 | 0.00 | 0.09 | 0.00 | 0.08 | 0.11 | 0.00 | 0.13 | 0.00 | 0.07 | 0.37 | 0.19 | 0.11 | 0.01 | 0.00 | 0.29 | 0.00 | 0.00 | 0.00 | 0.00 | 0.05 | 0.00 | 0.27 |
| 22:1ω9 | 0.30 | 0.32 | 0.31 | 0.38 | 0.73 | 0.54 | 0.30 | 0.21 | 0.53 | 0.70 | 1.74 | 0.44 | 0.62 | 0.35 | 0.51 | 0.30 | 0.31 | 0.23 | 0.47 | 0.22 | 0.59 | 0.37 |
| 22:1ω7 | 0.17 | 0.09 | 0.05 | 0.04 | 1.25 | 0.09 | 0.09 | 0.00 | 0.12 | 0.09 | 0.52 | 0.12 | 0.17 | 0.06 | 0.12 | 0.09 | 0.10 | 0.13 | 0.07 | 0.09 | 0.50 | 0.03 |
| 24:1ω9 | 1.35 | 1.15 | 0.68 | 0.39 | 10.63 | 0.45 | 1.04 | 0.52 | 0.73 | 0.31 | 2.53 | 0.84 | 1.11 | 0.52 | 0.43 | 0.84 | 0.65 | 1.10 | 0.59 | 0.92 | 5.11 | 0.45 |
| 24:1ω7 | 0.14 | 0.03 | 0.00 | 0.00 | 1.82 | 0.00 | 0.00 | 0.00 | 0.05 | 0.00 | 0.44 | 0.11 | 0.05 | 0.00 | 0.00 | 0.00 | 0.00 | 0.11 | 0.01 | 0.00 | 0.88 | 0.00 |
| 16:4 | 0.00 | 0.19 | 0.18 | 0.10 | 0.13 | 0.28 | 0.25 | 0.18 | 0.00 | 0.05 | 0.11 | 0.04 | 0.00 | 0.14 | 0.09 | 0.17 | 0.10 | 0.22 | 0.24 | 0.16 | 0.22 | 0.07 |
| 16:3 | 0.12 | 0.24 | 0.00 | 0.00 | 0.00 | 0.00 | 0.35 | 0.15 | 0.11 | 0.00 | 0.00 | 0.06 | 0.16 | 0.00 | 0.00 | 0.32 | 0.00 | 0.00 | 0.20 | 0.10 | 0.00 | 0.07 |
| 18:4ω3 | 0.00 | 0.00 | 0.00 | 0.00 | 0.02 | 0.05 | 0.12 | 0.00 | 0.03 | 0.04 | 0.08 | 0.08 | 0.00 | 0.05 | 0.08 | 0.00 | 0.00 | 0.04 | 0.00 | 0.05 | 0.00 | 0.00 |
| 18:2ω6 | 0.55 | 0.72 | 0.66 | 0.74 | 0.22 | 0.70 | 0.73 | 0.66 | 0.63 | 1.02 | 0.67 | 0.72 | 0.57 | 0.62 | 0.99 | 0.64 | 0.50 | 0.65 | 0.64 | 0.55 | 0.45 | 0.74 |
| 18:3ω3 | 0.15 | 0.00 | 0.00 | 0.00 | 0.18 | 0.00 | 0.00 | 0.00 | 0.08 | 0.00 | 0.00 | 0.11 | 0.07 | 0.05 | 0.10 | 0.00 | 0.00 | 0.04 | 0.04 | 0.00 | 0.10 | 0.00 |
| 20:4ω6 | 13.64 | 8.64 | 7.21 | 7.48 | 5.73 | 9.94 | 9.62 | 12.65 | 11.77 | 5.87 | 6.30 | 8.71 | 14.43 | 10.45 | 8.56 | 14.28 | 6.96 | 10.57 | 8.92 | 15.34 | 6.85 | 1.42 |
| 20:5ω3 | 1.81 | 1.61 | 0.99 | 1.10 | 0.26 | 1.18 | 0.88 | 2.18 | 2.56 | 0.09 | 1.17 | 1.61 | 1.59 | 0.21 | 2.32 | 2.01 | 0.24 | 1.56 | 0.85 | 2.12 | 0.16 | 3.53 |
| 20:3ω6 | 0.23 | 0.53 | 0.11 | 0.13 | 0.09 | 0.21 | 0.28 | 0.33 | 0.22 | 0.23 | 0.58 | 0.24 | 0.19 | 0.23 | 0.25 | 0.36 | 0.20 | 0.36 | 0.24 | 0.27 | 0.09 | 0.18 |
| 20:4ω3 | 0.14 | 0.25 | 0.07 | 0.08 | 0.13 | 0.14 | 0.05 | 0.16 | 0.19 | 0.18 | 0.12 | 0.21 | 0.12 | 0.16 | 0.15 | 0.16 | 0.15 | 0.18 | 0.18 | 0.13 | 0.12 | 1.12 |
| C20PUFA | 0.09 | 0.48 | 0.45 | 0.07 | 0.27 | 0.45 | 0.72 | 0.09 | 0.13 | 0.11 | 0.23 | 0.22 | 0.07 | 0.36 | 0.89 | 0.10 | 0.54 | 0.15 | 0.17 | 0.10 | 0.13 | 0.03 |
| 20:2ω6 | 0.17 | 0.30 | 0.20 | 0.26 | 0.12 | 0.38 | 0.27 | 0.28 | 0.27 | 0.24 | 0.25 | 0.23 | 0.19 | 0.36 | 0.32 | 0.19 | 0.17 | 0.19 | 0.29 | 0.17 | 0.19 | 0.15 |
| 22:5ω6 | 1.05 | 0.63 | 0.73 | 0.43 | 0.38 | 1.01 | 1.47 | 1.36 | 0.69 | 1.02 | 0.22 | 0.82 | 0.96 | 1.09 | 0.70 | 1.16 | 0.61 | 1.02 | 0.81 | 1.16 | 0.41 | 0.84 |
| 22:6ω3 | 19.92 | 11.78 | 10.71 | 12.48 | 4.01 | 13.19 | 17.52 | 22.75 | 23.28 | 15.18 | 2.39 | 14.43 | 20.04 | 18.21 | 14.50 | 21.01 | 9.88 | 17.96 | 15.85 | 21.00 | 7.73 | 24.13 |
| 22:4ω6 | 3.43 | 2.42 | 1.51 | 0.95 | 3.03 | 2.00 | 5.63 | 2.71 | 2.61 | 1.65 | 1.43 | 3.48 | 3.60 | 2.36 | 1.26 | 3.44 | 3.25 | 4.04 | 2.37 | 3.17 | 2.07 | 0.71 |
| 22:5ω3 | 3.30 | 0.10 | 1.59 | 1.56 | 0.44 | 2.14 | 2.24 | 3.18 | 4.84 | 3.43 | 1.06 | 4.03 | 2.87 | 3.44 | 2.56 | 3.71 | 2.25 | 3.80 | 3.05 | 2.98 | 1.90 | 7.13 |
| C22PUFA | 0.07 | 0.00 | 0.00 | 0.00 | 0.04 | 0.00 | 0.00 | 0.00 | 0.16 | 0.31 | 0.00 | 0.13 | 0.08 | 0.00 | 0.00 | 0.03 | 0.00 | 0.00 | 0.06 | 0.00 | 0.02 | 0.24 |
| 24:6ω3 | 0.06 | 0.00 | 0.00 | 0.03 | 0.11 | 0.00 | 0.00 | 0.07 | 0.08 | 0.06 | 0.00 | 0.09 | 0.04 | 0.00 | 0.00 | 0.00 | 0.00 | 0.09 | 0.00 | 0.00 | 0.21 | 0.27 |
| i14:0 | 0.00 | 0.00 | 0.00 | 0.00 | 0.00 | 0.00 | 0.00 | 0.00 | 0.00 | 0.00 | 0.00 | 0.11 | 0.01 | 0.00 | 0.00 | 0.00 | 0.13 | 0.00 | 0.00 | 0.00 | 0.02 | 0.00 |
| i15:0 | 0.22 | 0.31 | 0.28 | 0.34 | 0.61 | 0.36 | 0.41 | 0.20 | 0.15 | 0.36 | 0.95 | 0.25 | 0.26 | 0.17 | 0.32 | 0.26 | 0.44 | 0.21 | 0.14 | 0.25 | 0.45 | 0.30 |
| i16:0 | 0.26 | 0.01 | 0.20 | 0.32 | 0.32 | 0.29 | 0.00 | 0.00 | 0.10 | 0.18 | 0.61 | 0.33 | 0.12 | 0.19 | 0.30 | 0.00 | 0.34 | 0.23 | 0.16 | 0.08 | 0.41 | 0.00 |
| i17:0 | 1.15 | 0.99 | 0.81 | 0.99 | 1.64 | 1.45 | 0.69 | 0.78 | 0.79 | 0.94 | 1.49 | 0.80 | 0.63 | 0.83 | 0.90 | 0.83 | 0.53 | 0.79 | 0.88 | 0.69 | 2.01 | 0.73 |
| i18:0 | 0.07 | 0.12 | 0.12 | 0.05 | 0.37 | 0.76 | 0.48 | 0.04 | 0.09 | 0.06 | 0.76 | 0.11 | 0.04 | 0.63 | 0.06 | 0.08 | 0.44 | 0.05 | 0.39 | 0.02 | 0.16 | 0.33 |
| MBrFA | 0.31 | 0.23 | 0.40 | 0.20 | 0.28 | 0.06 | 0.00 | 0.04 | 0.32 | 0.20 | 0.00 | 0.11 | 0.30 | 0.07 | 0.26 | 0.08 | 0.32 | 0.26 | 0.10 | 0.16 | 0.24 | 0.23 |
| 16:0 FALD | 1.05 | 1.62 | 2.33 | 1.25 | 2.54 | 0.46 | 1.65 | 1.71 | 1.63 | 0.49 | 0.07 | 0.51 | 1.39 | 1.32 | 0.81 | 2.72 | 1.56 | 1.85 | 0.81 | 2.40 | 1.54 | 1.24 |
| 18:0 FALD | 0.22 | 0.36 | 0.50 | 0.38 | 2.99 | 0.30 | 0.20 | 0.22 | 0.30 | 0.14 | 0.45 | 0.19 | 0.27 | 0.26 | 0.20 | 0.32 | 0.44 | 0.30 | 0.20 | 0.31 | 1.01 | 0.14 |
