# Supplementary material for: Lipid, Fatty Acid and Energy Density Profiles of White Sharks: Insights into the Feeding Ecology and Ecophysiology of a Complex Top Predator
Source: PLoS One. 2014 May 28;9(5):e97877. doi: 10.1371/journal.pone.0097877 (PMC4037211; doi:10.1371/journal.pone.0097877)
Supplement: Table S2 — Liver full fatty acid profiles of individual white sharks and a single basking shark (BS1) analyzed in this study. (DOCX) [file pone.0097877.s002.docx]

**Table S2.** Liver full fatty acid profiles of individual white sharks and a single basking shark (BS1) analyzed in this study.

| Samples | 11d | 12b | 14c | 15b | 16b | 17b | 19b | BS1b |
| --- | --- | --- | --- | --- | --- | --- | --- | --- |
| 14:0 | 2.61 | 2.09 | 5.42 | 2.81 | 3.05 | 2.19 | 4.53 | 6.76 |
| 15:0 | 0.54 | 0.39 | 0.50 | 0.59 | 0.67 | 0.53 | 0.94 | 0.85 |
| 16:0 | 17.90 | 19.72 | 17.73 | 18.19 | 20.65 | 17.72 | 21.57 | 14.92 |
| 17:0 | 0.74 | 0.69 | 0.59 | 0.82 | 0.96 | 0.71 | 0.97 | 0.71 |
| 18:0 | 6.71 | 7.90 | 5.04 | 6.46 | 7.21 | 6.90 | 5.95 | 2.80 |
| 19:0 | 0.15 | 0.13 | 0.12 | 0.16 | 0.14 | 0.14 | 0.24 | 0.08 |
| 20:0 | 0.16 | 0.09 | 0.15 | 0.20 | 0.17 | 0.16 | 0.22 | 0.16 |
| 22:0 | 0.12 | 0.08 | 0.06 | 0.17 | 0.10 | 0.08 | 0.11 | 0.11 |
| 24:0 | 0.04 | 0.05 | 0.03 | 0.05 | 0.05 | 0.05 | 0.05 | 0.03 |
| 16:1ω9 | 0.49 | 0.73 | 0.42 | 0.45 | 0.49 | 0.54 | 0.75 | 0.10 |
| 16:1ω7 | 5.10 | 4.04 | 16.70 | 8.33 | 8.15 | 4.72 | 11.23 | 1.29 |
| 17:1ω8+a17:0 | 0.75 | 0.79 | 0.85 | 0.93 | 0.87 | 0.80 | 1.33 | 0.25 |
| 17:1 | 0.05 | 0.08 | 0.06 | 0.03 | 0.03 | 0.06 | 0.04 | 0.06 |
| 18:1ω9 | 16.69 | 18.77 | 22.05 | 19.64 | 16.91 | 18.80 | 19.87 | 5.07 |
| 18:1ω7 | 5.87 | 7.64 | 7.51 | 5.45 | 5.88 | 5.70 | 4.93 | 2.01 |
| 18:1ω5 | 0.24 | 0.27 | 0.28 | 0.26 | 0.18 | 0.28 | 0.22 | 0.45 |
| 19:1 | 0.24 | 0.33 | 0.19 | 0.27 | 0.25 | 0.25 | 0.24 | 0.29 |
| 20:1ω11 | 0.59 | 0.54 | 0.07 | 0.52 | 0.36 | 0.66 | 0.76 | 0.00 |
| 20:1ω9 | 3.03 | 4.66 | 1.70 | 2.79 | 1.97 | 3.57 | 1.50 | 28.23 |
| 20:1ω7 | 0.42 | 0.35 | 0.43 | 0.40 | 0.46 | 0.50 | 0.36 | 0.55 |
| 22:1ω11 | 0.56 | 0.44 | 0.25 | 0.45 | 0.29 | 0.64 | 0.30 | 15.96 |
| 22:1ω9 | 0.49 | 0.65 | 0.29 | 0.42 | 0.37 | 0.76 | 0.35 | 1.00 |
| 22:1ω7 | 0.14 | 0.00 | 0.08 | 0.09 | 0.09 | 0.08 | 0.11 | 0.94 |
| 24:1ω9 | 0.30 | 0.27 | 0.22 | 0.33 | 0.29 | 0.45 | 0.39 | 0.00 |
| 24:1ω7 | 0.05 | 0.00 | 0.04 | 0.05 | 0.06 | 0.06 | 0.06 | 0.11 |
| 16:4 | 0.12 | 0.00 | 0.06 | 0.12 | 0.14 | 0.09 | 0.07 | 0.00 |
| 16:3 | 0.17 | 0.13 | 0.10 | 0.19 | 0.23 | 0.21 | 0.10 | 0.92 |
| 18:4ω3 | 0.57 | 0.13 | 0.34 | 0.60 | 0.57 | 0.56 | 0.33 | 1.07 |
| 18:2ω6 | 1.24 | 0.97 | 1.36 | 1.24 | 1.07 | 1.22 | 1.03 | 0.36 |
| 18:3ω3 | 0.38 | 0.00 | 0.31 | 0.43 | 0.37 | 0.54 | 0.33 | 0.26 |
| 20:4ω6 | 2.38 | 1.81 | 1.09 | 2.04 | 2.08 | 1.86 | 1.78 | 0.26 |
| 20:5ω3 | 4.27 | 1.66 | 2.09 | 2.98 | 2.92 | 3.75 | 1.83 | 1.78 |
| 20:3ω6 | 0.17 | 0.16 | 0.21 | 0.16 | 0.17 | 0.15 | 0.13 | 0.08 |
| 20:4ω3 | 0.48 | 0.28 | 0.38 | 0.54 | 0.37 | 0.44 | 0.51 | 1.63 |
| C20PUFA | 0.15 | 0.10 | 0.15 | 0.19 | 0.22 | 0.12 | 0.25 | 0.00 |
| 20:2ω6 | 0.30 | 0.18 | 0.24 | 0.35 | 0.34 | 0.30 | 0.33 | 0.25 |
| 22:5ω6 | 0.27 | 0.83 | 0.50 | 0.02 | 1.00 | 0.55 | 0.58 | 0.32 |
| 22:6ω3 | 16.91 | 14.54 | 4.47 | 13.26 | 11.83 | 16.46 | 7.06 | 6.29 |
| 22:4ω6 | 1.03 | 0.95 | 0.80 | 1.07 | 1.44 | 0.77 | 0.92 | 0.75 |
| 22:5ω3 | 4.35 | 4.47 | 2.67 | 3.34 | 3.99 | 3.32 | 1.94 | 0.10 |
| C22PUFA | 0.30 | 0.23 | 0.15 | 0.17 | 0.18 | 0.14 | 0.17 | 0.11 |
| 24:6w3 | 0.16 | 0.12 | 0.07 | 0.16 | 0.15 | 0.14 | 0.14 | 0.15 |
| i14:0 | 0.04 | 0.01 | 0.62 | 0.15 | 0.18 | 0.06 | 0.44 | 0.00 |
| i15:0 | 0.27 | 0.30 | 0.48 | 0.31 | 0.26 | 0.22 | 1.21 | 0.36 |
| i16:0 | 0.21 | 0.21 | 0.47 | 0.30 | 0.24 | 0.27 | 0.91 | 0.14 |
| i17:0 | 0.54 | 0.68 | 0.52 | 0.52 | 0.56 | 0.51 | 0.71 | 0.20 |
| i18:0 | 0.16 | 0.10 | 0.24 | 0.29 | 0.29 | 0.16 | 0.25 | 0.45 |
| MBrFA | 0.04 | 0.12 | 0.17 | 0.04 | 0.23 | 0.18 | 0.21 | 0.14 |
| 16:0 FALD | 0.51 | 0.55 | 0.31 | 0.56 | 0.53 | 0.52 | 0.49 | 0.56 |
| 18:0 FALD | 0.14 | 0.10 | 0.12 | 0.16 | 0.13 | 0.12 | 0.20 | 0.07 |
